# Pluralibacter gergoviae: an uncommon pathogen in peritoneal dialysis-related peritonitis: the second documented case worldwide

**DOI:** 10.1093/omcr/omaf219

**Published:** 2025-10-29

**Authors:** Bahaa Arafat, Mohammed Dibas, Baraa Emran, Ahmed Salous, Ahmed Enaya, Alaa Sarsour, Zakaria Hamdan, Noor Abulehia

**Affiliations:** Department of Medicine, Faculty of Medicine and Health Sciences, An-Najah National University, Nablus P304, Palestine; Department of Medicine, Faculty of Medicine and Health Sciences, An-Najah National University, Nablus P304, Palestine; Department of Medicine, Faculty of Medicine and Health Sciences, An-Najah National University, Nablus P304, Palestine; Department of Medicine, Faculty of Medicine and Health Sciences, An-Najah National University, Nablus P304, Palestine; Department of Internal Medicine, An-Najah National University Hospital, Nablus P304, Palestine; Kidney and Dialysis Department, An-Najah National University Hospital, Nablus P304, Palestine; Department of Internal Medicine, An-Najah National University Hospital, Nablus P304, Palestine; Kidney and Dialysis Department, An-Najah National University Hospital, Nablus P304, Palestine

**Keywords:** peritonitis, spontaneous bacterial peritonitis, peritoneal dialysis, Pluralibacter gergoviae, end-stage renal disease

## Abstract

Peritonitis remains a serious complication in patients undergoing peritoneal dialysis, often caused by common organisms such as *Staphylococcus aureus*. However, rare pathogens may also play a role. We report a case of peritonitis in a 41-year-old female on continuous ambulatory peritoneal dialysis, in whom Pluralibacter gergoviae was identified as the causative organism. The patient presented with abdominal pain and cloudy dialysate but remained hemodynamically stable. Peritoneal fluid analysis revealed elevated white cell counts, and culture Pluralibacter gergoviae, which was sensitive to multiple antibiotics. Initial empiric therapy was adjusted accordingly, leading to clinical and laboratory improvement. This is the second reported case of Pluralibacter gergoviae-related peritonitis in the literature. This case underscores the importance of recognizing uncommon pathogens in peritoneal dialysis-related infections and the role of prompt microbiological diagnosis in guiding targeted therapy. Increased awareness and reporting of such cases are essential to enhance understanding and optimize patient management.

## Introduction

Peritoneal dialysis (PD) is a widely used renal replacement therapy for patients with end-stage renal disease (ESRD) as it carries several benefits, including home-based management [1]. However, PD is associated with various complications, the most serious of which is peritonitis. PD-related peritonitis is a major cause of morbidity, accounting for approximately 8.6% of total deaths among PD patients [2].

The majority of PD-related peritonitis cases are caused by Gram-positive bacteria, while less common, Gram-negative bacteria such as *Escherichia coli* and *Pseudomonas* species are also implicated in PD peritonitis and are associated with more severe clinical outcomes [3, 4]. Rare organisms have also been identified in isolated cases. One such uncommon pathogen is *Pluralibacter gergoviae*.

Pluralibacter gergoviae, is an environmental Gram-negative bacillus that has been previously identified in cases of dental plaque colonization and nosocomial outbreaks [5, 6]. However, it’s not widely recognized as a causative agent in PD-related peritonitis with the first reported case being in 2017 [7]. We report the second documented case of Pluralibacter gergoviae causing peritoneal dialysis-associated peritonitis, with valuable insights into its clinical presentation, microbiological identification, and therapeutic management.

## Case presentation

A 41-year-old female patient with a known history of end-stage renal disease (ESRD) on continuous ambulatory peritoneal dialysis (CAPD) presented to the emergency department with vague abdominal pain for 12 h. Pain was diffuse, gradual in onset, non-radiating, and not associated with vomiting or bowel changes. The patient’s medical history includes Diabetes mellitus for 30 years, which was complicated by diabetic nephropathy and hypertension for 11 years. The patient has no previous history of peritonitis incidents. The patient is on CAPD using a combination of 1.5% and 2.5% dextrose solutions, with a total daily volume of 2 liters.

On examination, the patient was hemodynamically stable, with a blood pressure of 126/77 mmHg, heart rate of 96 bpm, respiratory rate of 21 breaths per minute, temperature of 37.8°C, and oxygen saturation of 97%. Abdominal examination revealed mild diffuse tenderness without guarding or rigidity. The peritoneal dialysis catheter exit site was clean, with no signs of infection. However, the peritoneal fluid appeared turbid.

Peritoneal dialysis fluid analysis and standard peritoneal fluid culture techniques were performed. The analysis showed a total nucleated cell count of 1124 cells/μL, including a WBC count of 1100 cells/μL and an RBC count of 50 cells/μL. Additional laboratory investigations were conducted, as summarized in Table 1.

**Table 1:** Laboratory investigations at admission.

Laboratory test	Result	Normal ranges
WBC	11.1 × 10^3^/μL	4.5–11 × 10^3^/μL
Albumin	3.70 g/dL	3.5–5.5 g/dL
BUN	52.36 mg/dL	7–18 mg/dL
Creatinine	6.92 mg/dL	0.6–1.2 mg/dL
Calcium	8.83 mg/dL	8.4–10.2 mg/dL
Phosphate	5.44 mg/dL	3.0–4.5 mg/dL
Potassium	5.11 mmol/L	3.5–5.0 mmol/L
Sodium	132.40 mmol/L	136–146 mmol/L
CRP	20 mg/dl	Less than 0.3 mg/dL

The patient was started on an empiric antibiotic regimen, which included a 1 g loading dose of, vancomycin and an 80 mg loading dose of gentamicin, both administered intravenously. The following day, the patient received a 6 g loading dose of ceftazidime and a 6 g loading dose of cefazolin, also given intravenously. Seventy-two hours later, a peritoneal fluid culture revealed Gram-negative Pluralibacter gergoviae. Sensitivity results are summarized in Table 2.

**Table 2:** Culture sensitivity results of the peritoneal fluid sample.

Sensitivity	Antibiotic
Sensitive	Amoxicillin/Clavulanic Acid
Sensitive	Ceftazidime
Sensitive	Ceftriaxone
Sensitive	Cefepime
Sensitive	Ciprofloxacin
Sensitive	Gentamicin
Sensitive	Levofloxacin
Sensitive	Meropenem
Sensitive	Imipenem
Sensitive	Piperacillin/Tazobactam
Sensitive	Trimethoprim/Sulfamethoxazole

Repeated peritoneal fluid analysis on the fourth day revealed a WBC count of 130 cells/μL and an RBC count of 50 cells/μL with a negative peritoneal fluid culture. Additionally, the peritoneal fluid became less turbid, indicating an effective response to treatment. Consequently, the antibiotic regimen was adjusted to ceftazidime and cefazolin.

**Fig. 1:**
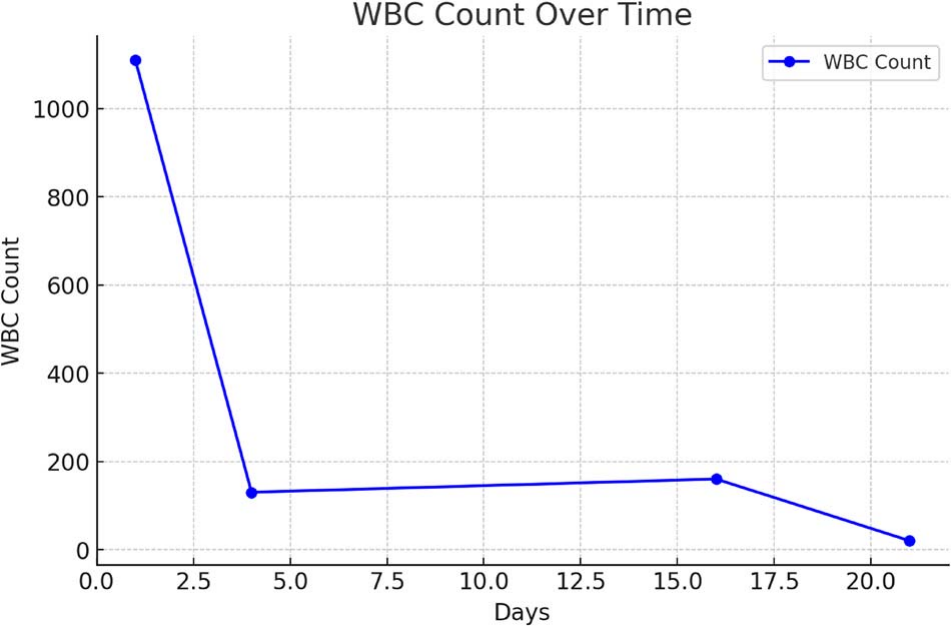
Describes changes in peritoneal fluid WBC count over treatment period. Data are presented as absolute peritoneal fluid WBC count over time. The WBC count was measured at multiple time points. A sharp decline was observed after day 1, followed by stabilization and a subsequent decrease toward day 21.

After two weeks the patient presented with vague abdominal pain, laboratory work showed peritoneal fluid WBC count of 160 cells/μL and an RBC count of 10 cells/μL with a negative peritoneal fluid culture. As a result, the patient was admitted for intraperitoneal administration of an 8 g dose of ceftazidime. On follow-up, the peritoneal fluid color had normalized, peritoneal fluid WBC count had was 20 cells/μL, and the patient’s symptoms had completely resolved. Figure 1 summarizes the changes in peritoneal fluid WBC count throughout the treatment period.

## Discussion

Pluralibacter gergoviae is a Gram-negative, rod-shaped organism first isolated from urinary samples in 1976. This organism has been implicated in several outbreaks, particularly in neonatal intensive care units (NICUs). Beyond healthcare settings, P. gergoviae is found in various environmental sources, including sewage, food, and oils, raising concerns about its role in nosocomial and community-acquired infections [7, 8].

Peritonitis continues to be a major complication, leading to increased morbidity and mortality in peritoneal dialysis patients. Various organisms are implicated in PD-related peritonitis, with *Staphylococcus aureus* and Streptococci being the most frequently identified pathogens [9]. However, in this case, Pluralibacter gergoviae was identified by laboratory culture. This finding raises concerns about the role of uncommon pathogens in peritonitis among peritoneal dialysis patients.

Diagnosis of Pluralibacter gergoviae peritonitis relies on integrating clinical presentation, peritoneal fluid analysis, and culture results. In this case, the patient presented with classic signs of peritonitis, including abdominal pain and cloudy peritoneal fluid. However, the absence of systemic manifestations, such as fever or marked leukocytosis, highlighted the importance of rapid and precise microbiological confirmation to ensure an accurate diagnosis [10].

According to the 2022 update of the International Society for Peritoneal Dialysis (ISPD) guidelines, empirical treatment of (PD)-associated peritonitis should provide broad-spectrum coverage This is achieved by combining a first-generation cephalosporin or vancomycin for Gram-positive coverage with a third-generation cephalosporin or an aminoglycoside for Gram-negative coverage. The intraperitoneal (IP) route is preferred due to its ability to achieve high local antibiotic concentrations, The typical treatment duration is 21 days [11].

Antibiotics were adjusted based on culture results and sensitivities for targeted treatment. Adjustments in the route of antibiotic administration during the treatment period highlight the superiority of intraperitoneal (IP) over intravenous delivery in managing peritonitis. There is limited data concerning the patterns of antimicrobial resistance in the context of PD-related peritonitis throughout the Middle East, with no thorough regional studies existing as a point for direct comparison. This highlights the significance of such case, which involves a rare pathogen causing peritonitis in a PD patient, that adds to the sparse regional literature and underscores the need for broader surveillance and reporting.

## Conclusion

This case, although rare, emphasizes the importance of considering Pluralibacter gergoviae as a possible pathogen in peritonitis associated with PD. There has to be precise culture techniques followed by molecular techniques if needed to identify rare organisms Given the rarity of this pathogen. Early recognition, coupled with prompt initiation of targeted antibiotic therapy, remains critical for preventing complications and avoiding catheter loss. As this represents the second documented case worldwide, further case reports and surveillance are essential to have a better understanding of the resistance pattern if there is one and the epidemiology of it.
